# Editorial: Respiratory dysfunction in neurological disease and injury: novel mechanisms and potential therapeutics

**DOI:** 10.3389/fphys.2025.1554396

**Published:** 2025-01-31

**Authors:** Savannah Lusk, Robert M. Wadolowski, Mai K. ElMallah, Carlos B. Mantilla, Teresa Pitts, Irene C. Solomon

**Affiliations:** ^1^ Department of Neuroscience, Baylor College of Medicine, Houston, TX, United States; ^2^ Department of Physiology and Biophysics, Renaissance School of Medicine, Stony Brook University, Stony Brook, NY, United States; ^3^ Departments of Pediatrics, Cell Biology, and Neurobiology, Duke University, Durham, NC, United States; ^4^ Department of Anesthesiology and Perioperative Medicine, Mayo Clinic, Rochester, NY, United States; ^5^ Department of Physiology and Biomedical Engineering, Mayo Clinic, Rochester, NY, United States; ^6^ Department of Speech, Language and Hearing Sciences, University of Missouri, Columbia, MO, United States

**Keywords:** breathing in disease, neurodegenerative respiratory pathology, developmental respiratory pathology, motoneuron and muscular respiratory pathology, disordered breathing, respiratory therapeutics

**FIGURE 1:**
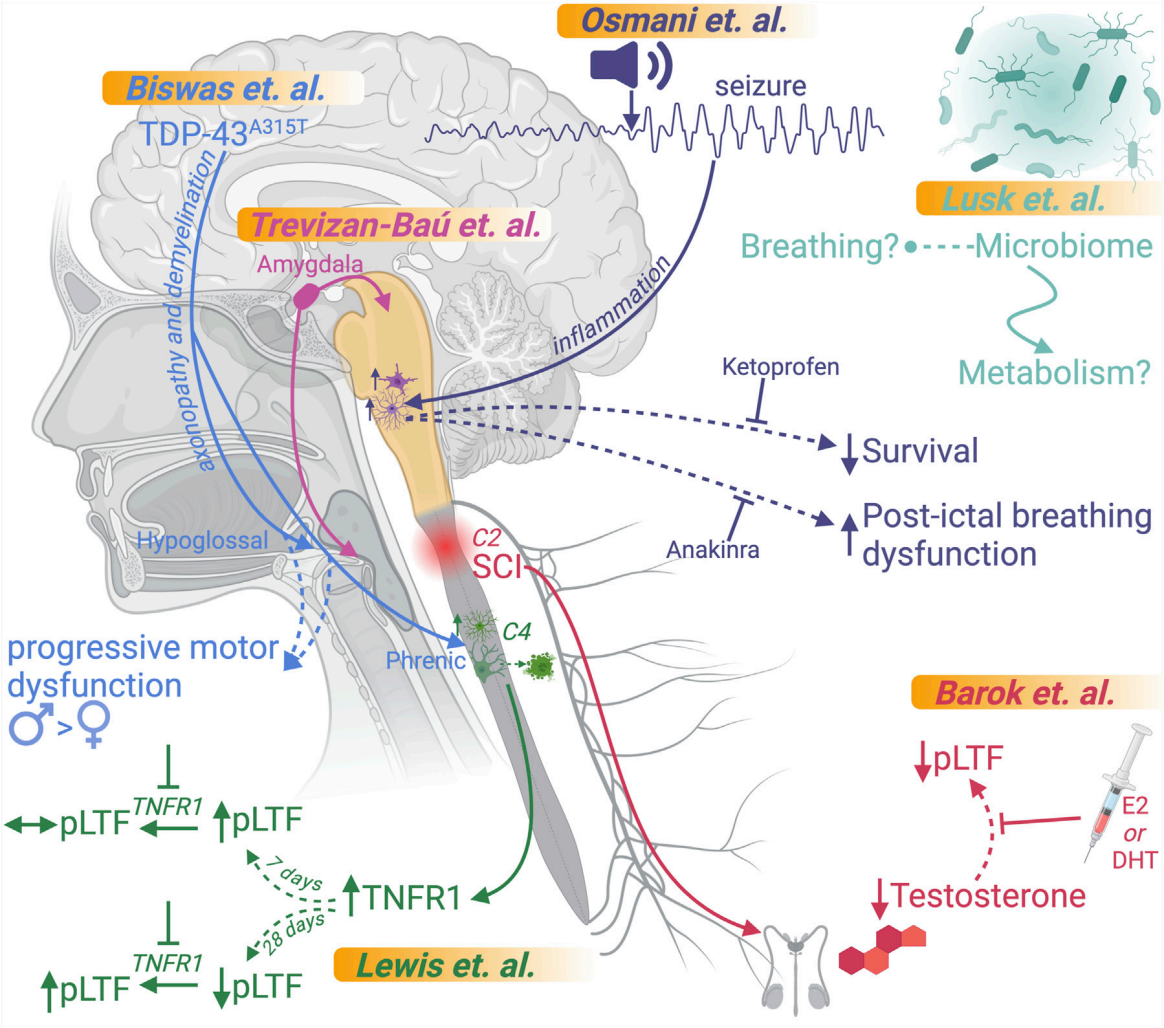
Overview of articles in this Research Topic. Graphical representations of the findings of each article submitted to this Research Topic are included. This figure was created using BioRender.

Understanding the intricate mechanisms governing respiration is pivotal for addressing the diverse and complex conditions that disrupt this essential function. Over the past 2 decades, significant advancements in our understanding of respiratory networks have opened new avenues for discovery, enabling a deeper exploration of diseases with overlapping etiologies and trajectories and promoting a deeper appreciation of relationships between neurological, developmental, and injury-induced respiratory dysfunction. With a focus on control of breathing, this Research Topic was developed and organized to highlight recent advances in various neurological- and injury-based models for respiratory dysfunction, emphasizing mechanisms of dysfunction and potential therapeutic approaches aimed at ameliorating dysfunction (Figure 1).

Understanding basic neural circuit connectivity that regulates respiratory (dys)function is an essential first step. While brainstem nuclei have long been recognized as central components of respiratory maintenance, additional CNS sites have recently been implicated in higher-level modulation. In the review by Trevizan-Baú et al., the authors explore the role of the amygdala, a key brain region in the temporal lobe, in regulating respiratory functions, including breathing, swallowing, coughing, airway smooth muscle contraction, and mucus secretion (Trevizan-Baú et al.) The article also examines how amygdala dysfunction may contribute to respiratory and airway pathologies, such as those seen in anxiety disorders and airway diseases.

Beyond the basic circuitry involved, mechanistic insights and therapeutic approaches necessitate discovery in the disease state. In the study by Barok et al., the authors first confirm that circulating testosterone, a critical sex steroid, is reduced for at least 2 weeks following spinal cord injury (SCI) and then investigate the role of supplemental dihydrotestosterone (DHT) and 17β-estradiol (E2) on respiratory function and neuroplasticity in a rat model of cervical C2-hemisection SCI (Barok et al.). Their study demonstrated that both DHT and E2 supplementation restore the expression of phrenic long-term facilitation (pLTF), a respiratory neuroplasticity phenomenon, and improve breathing recovery, and that E2-treated rats exhibit enhanced ventilation and normalized metabolic function. These findings suggest that steroidal hormones may be key therapeutic agents for improving respiratory motor recovery in the subacute phase of SCI.

But, of course, respiratory regulation is more complicated than hormonal signaling modifications. In the study by Osmani et al., the authors investigate contributions of neuroinflammation in respiratory dysfunction and mortality associated with repeated seizures using a genetic rat model (SS^kcnj16−/−^) that mimics key features of Sudden Unexpected Death in Epilepsy (SUDEP) (Osmani et al.). Their study revealed that repeated audiogenic seizures triggered neuroinflammation in brainstem regions critical for respiratory control, noting specifically an increase in proinflammatory cytokines IL-1α and IL-1β and evidence of microglial activation in the pre-Bötzinger complex and nucleus ambiguus after 3–5 days of seizures. They also showed that treatment with the IL-1 receptor antagonist anakinra mitigated progressive post-ictal ventilatory suppression but did not prevent seizure-related mortality. In contrast, treatment with the nonsteroidal anti-inflammatory drug ketoprofen exacerbated post-ictal breathing dysfunction but completely prevented mortality. The findings highlight a complex interplay between inflammation and respiratory control mechanisms in the context of epilepsy and suggest that distinct inflammatory pathways may independently drive ventilatory impairment and seizure-related mortality.

Additional roles for inflammation were noted in work by Lewis et al., who investigate the involvement of tumor necrosis factor-alpha (TNF-α) and its receptor (TNFR1) in phrenic motor neuron loss and compensatory mechanisms using a rat model of selective motor neuron death induced by cholera toxin B conjugated to saporin (CTB-SAP) to mimic aspects of neurodegenerative diseases like amyotrophic lateral sclerosis (ALS) and spinal muscular atrophy (Lewis et al.). Their study demonstrated increased TNFR1 expression on phrenic motor neurons and reactive astrocytic morphology and increased astrocyte numbers in the phrenic motor nucleus at 28 days post-injection (28d CTB-SAP). It revealed that pLTF was attenuated in rats at 7 days post-injection (7d CTB-SAP) and enhanced in rats at 28d CTB-SAP when treated with an inhibitor for TNFR1 (sTNFR1i). While differences in TNF-α signaling in early stage (7d CTB-SAP) and later stage (28d CTB-SAP) motor neuron loss were identified, the findings suggest that TNFR1 antagonism could serve as a potential therapeutic strategy to improve respiratory motor neuron function, compensatory plasticity, and breathing quality in late-stage respiratory motor neuron diseases.

Aberrant protein expression and signaling have also been shown to result in neuroinflammation within respiratory networks, and in the study by Biswas et al., the authors used the Prp-TDP-43^A315T^ mouse model of ALS to explore the impact of TDP-43 mutations on respiratory function and respiratory motor neuron pathology (Biswas et al.). Their study revealed that male TDP-43^A315T^ mice exhibited early-onset, rapid disease progression and premature death (∼9.5 weeks). In comparison, female TDP-43^A315T^ mice showed delayed onset (∼23 weeks) with rapid progression at later stages. Respiratory deficits in males included reduced tidal volume, minute ventilation, and erratic breathing patterns at rest and during respiratory challenges, while females exhibited less pronounced deficits at rest but significant impairments during hypoxic and hypercapnic stress. Substantial motor neuron loss was accompanied by heightened microglial and astrocytic activation, indicative of neuroinflammation in both the hypoglossal and phrenic motor nuclei, with axonopathy and demyelination also noted. The study demonstrates that the TDP-43^A315T^ mouse model recapitulates the significant neuropathology and respiratory deficits seen in ALS patients with the A315T mutation in TDP-43, suggesting that this model may serve as a valuable tool for investigating ALS-related respiratory deficits and testing novel therapies.

Finally, there is a growing interest in peripheral signaling from the gut and its role in respiratory regulation in health and disease. In the study by Lusk et al., the authors use neonatal and adult germ-free (GF) mice, which lack a microbiome, and specific pathogen-free (SPF) mice with an intact microbiome to investigate the relationship between respiratory function and the microbiome. Their study found that compared to SPF neonates, GF neonates display altered baseline respiratory parameters, which include increased tidal volume and decreased heart rate, as well as recovery cardiorespiratory parameters that include shorter induction times, faster recovery of heart rate, and altered gasp patterns during anoxic challenges albeit no significant effects on survival or autoresuscitation outcomes were noted. In adults, baseline respiratory measures were largely unaffected. However, GF mice exhibited a reduction in oxygen consumption that could be recovered by fecal material transplant, suggesting an influence of the microbiome on metabolic demand. Interestingly, GF mice had fewer apneas and sighs during respiratory challenges, indicating potentially reduced respiratory adaptability. While the absence of a microbiome altered specific respiratory and metabolic parameters, it did not significantly impair overall survival or respiratory function, which appears to contrast with some observations from prior studies using antibiotic depletion to disrupt the gut microbiome. Their findings suggest that microbiome effects on respiratory outcomes may be secondary to changes in body composition, metabolism, and autonomic control.

This Research Topic seeks to consolidate cutting-edge research that not only elucidates the baseline functioning of respiratory networks but also addresses the underlying mechanisms of respiratory dysfunction across a spectrum of conditions. By showcasing studies on neurodegenerative disorders such as ALS, developmental anomalies like sudden infant death syndrome (SIDS) and SUDEP, and neural injuries including SCI, this collection aims to foster a more comprehensive understanding of how respiration is impacted in health and disease and uncover actionable strategies that address respiratory dysfunction. The insights garnered here can redefine our approach to respiratory health and catalyze future breakthroughs in preventing and mitigating the devastating impacts of respiratory dysfunction.

